# An Effective and Interpretable Sleep Stage Classification Approach Using Multi-Domain Electroencephalogram and Electrooculogram Features

**DOI:** 10.3390/bioengineering12030286

**Published:** 2025-03-13

**Authors:** Xin Xu, Bei Zhang, Tingting Xu, Junyi Tang

**Affiliations:** School of Communication and Information Engineering, Nanjing University of Posts and Telecommunications, Nanjing 210003, China; xuxin@njupt.edu.cn (X.X.); zb568104848@163.com (B.Z.); b23012214@njupt.edu.cn (J.T.)

**Keywords:** sleep staging, EEG, feature extraction, feature selection, XGBoost

## Abstract

Accurate sleep staging is critical for assessing sleep quality and diagnosing sleep disorders. Recent research efforts on automated sleep staging have focused on complex deep learning architectures that have achieved modest improvements in classification accuracy but have limited real-world applicability due to the complexity of model training and deployment and a lack of interpretability. This paper presents an effective and interpretable sleep staging scheme that follows a classical machine learning pipeline. Multi-domain features were extracted from preprocessed electroencephalogram (EEG) signals, and novel electrooculogram (EOG) features were created to characterize different sleep stages. A two-step feature selection strategy combining F-score pre-filtering and XGBoost feature ranking was designed to select the most discriminating feature subset, which was then fed into an XGBoost model for sleep stage classification. Through a rigorous double-cross-validation procedure, our approach achieved competitive classification performance on the public Sleep-EDF dataset (accuracy 87.0%, F1-score 86.6%, Kappa coefficient 0.81) compared with the state-of-the-art deep learning methods and provided interpretability through feature importance analysis. These promising results demonstrate the effectiveness of the proposed sleep staging model and show its potential in practical applications due to its low complexity, interpretability, and transparency.

## 1. Introduction

Quality sleep is fundamental to human health. In contrast, sleep disorders have been shown to be closely associated with a variety of brain diseases [1]. Therefore, the accurate assessment of sleep quality is of great value for promoting physical and mental health and preventing and diagnosing brain diseases.

Currently, the gold standard for sleep quality assessment is polysomnography (PSG), which consists of an electroencephalogram (EEG), an electrooculogram (EOG), and other physiological signals including muscle activity, heart rhythm, and respiration. The PSG data are usually segmented into 30 s epochs, and are then classified into Wake (W), Rapid Eye Movement (REM), and Non-REM (N1, N2, N3, and N4) stages for further analysis, according to either the American Academy of Sleep Medicine (AASM) standard [2] or the Rechtschaffen and Kales (R and K) standard [3]. This process, known as sleep staging or sleep stage classification, is time-consuming, labor-intensive, and highly dependent on the expertise of the physician.

Many research efforts have been made to automate the sleep staging process. Early works followed the classical machine learning pipeline that includes feature engineering and the training of classical machine learning models. For example, Alickovic et al. proposed an automated sleep stage classification method based on single-channel EEG signals using an ensemble Support Vector Machine (SVM). The method achieved an 84.46% classification accuracy, but the high complexity of model training limits its application in lightweight devices [4]. Rahman et al. proposed a sleep stage classification method based on single-channel EOG signals, utilizing statistical features in the Discrete Wavelet Transform (DWT) domain and classifying them using RUSBoost, Random Forest, and SVM. The experimental results showed higher accuracy in N1 stage classification compared to traditional EOG and EEG methods. However, the classification performance for other stages was limited [5]. Overall, the performance of conventional machine learning methods depends on the representativeness of the extracted features, which requires certain domain knowledge.

In the past few years, research in this area has shifted towards designing more sophisticated deep learning architectures that automatically learn hierarchical representations from raw data by integrating multiple layers of linear and non-linear processing units. For example, Supratak et al. proposed a deep learning model named DeepSleepNet for automatic sleep stage scoring based on a raw single-channel EEG, without using any handcrafted features [6]. The model employs Convolutional Neural Networks (CNNs) to extract time-invariant features and utilizes Bidirectional Long Short-Term Memories (LSTMs) to learn transition rules between sleep stages from EEG epochs using a two-step training algorithm. Emadeldeen et al. introduced a novel attention-based deep learning architecture called AttnSleep, which includes a multi-resolution CNN module for feature extraction and a temporal context encoder to capture the temporal dependencies in the extracted features [7]. Overall, the state-of-the-art deep learning models have shown improved classification performance by utilizing complex neural network architectures. However, the improvement over conventional machine learning approaches is not pronounced.

Deep learning-based solutions skip the step of manual feature extraction but generally suffer from high model complexity and poor transparency, making them difficult to deploy and generalize in real-world applications. A few works have tried to design light-weight deep learning models, and these reported similar performance compared to complex deep architectures [8]. Recent research also shows that classical machine learning models with expressive features are able to achieve competitive classification performance compared to deep learning for sleep-scoring tasks and provide interpretability that is crucial for medical research [9]. Furthermore, although small incremental improvements have been consistently reported, the classification accuracy of current staging solutions still needs to be further improved. For example, the classification accuracy for the N1 stage reported by state-of-the-art deep learning algorithms is below 50%, which is not satisfactory for clinical applications [10]. These results together suggest the need for efficient, lightweight, and interpretable machine learning solutions that are acceptable in practical sleep staging applications.

In this work, we designed an effective and interpretable machine learning scheme for automatic sleep staging. The main contributions of this work are as follows:

1. We extracted multi-domain features from single-channel EEG signals that capture well the spectral and temporal characteristics of different sleep stages. We also proposed two novel EOG features that significantly improve the classification accuracy of the N1 and REM stages.
2. We designed a novel two-step feature selection algorithm combining F-score prefiltering and XGBoost feature ranking that effectively identifies a small subset of discriminating features for sleep stage classification. This lays the foundation for the continued incorporation of new features in future works. The feature analysis results also provided quantifiable information for understanding the differences between sleep stages.
3. We validated the proposed scheme on the popular Sleep-EDF database containing PSG data from 150 subjects following strict double cross-validation procedures and compared the results with state-of-the-art deep learning models. We showed that competitive performance can be achieved with a small number of representative features using an interpretable machine learning model.

The remainder of this paper is organized as follows: Section 2 presents the proposed sleep staging method, including the dataset, preprocessing, feature extraction, feature selection, and the design of the classification model. Section 3 presents the experiments and the results, covering evaluation methods, classification results, and feature analysis. Section 4 discusses the effectiveness of the method, the impact of feature selection, and the performance across different sleep stages. Finally, Section 5 summarizes the findings and outlines directions for future research.

## 2. Proposed Method

**Figure 1:**
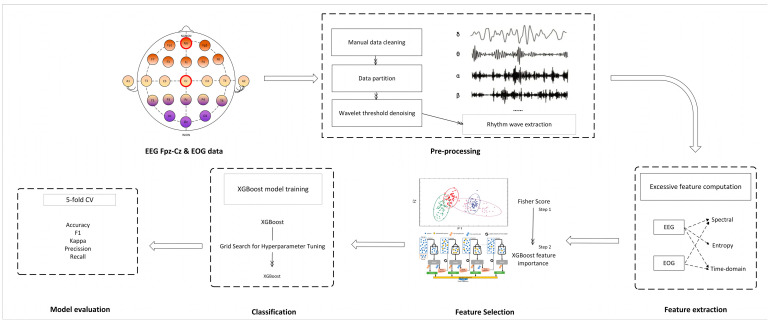
Flow chart of automatic sleep EEG staging.

The proposed sleep stage classification scheme includes six main steps: EEG and EOG data acquisition, data preprocessing, feature extraction, feature selection, classification, and performance evaluation. EEG and EOG data are prefiltered and denoised, from which a comprehensive EEG feature set is constructed and combined with innovative EOG features to represent the characteristics of different sleep stages. A two-step feature selection strategy is designed to select the most discriminating feature subset with reduced dimensionality. Finally, an XGboost model is trained for sleep stage classification, and the performance is evaluated and compared with state-of-the-art methods. Figure 1 shows the schematic flow chart of the proposed sleep stage classification approach. The details of each step are described as follows.

### 2.1. Dataset

We evaluated the performance of the proposed sleep staging approach using the public Sleep-EDF dataset [11]. The dataset contains two sets of subjects from two studies: age effects in healthy subjects (SC) and temazepam effects on sleep (ST). We used 150 PSG recordings from 75 subjects in the SC data, which included electroencephalogram (EEG, with Fpz-Cz) and EOG data. Both the EEG and EOG signals were sampled at 100 Hz, and each subject had two nights of complete PSG recordings. Note that subjects 13, 36, and 52 were excluded due to one missed night of PSG recordings.

The PSG recordings were segmented into 30 s epochs and were manually classified into one of the eight classes (W, N1, N2, N3, N4, REM, MOVEMENT, and UNKNOWN) by sleep experts according to the R and K standard [11]. Following existing methods, we excluded MOVEMENT and UNKNOWN epochs and merged the N3 and N4 stages as a single N3 stage to represent deep sleep. In addition, the original PSG recordings contained long periods of awake state before the start and after the end of the sleep period, and as such, only 30 min before and after the sleep periods were kept for the following experiments. The number of 30 s epochs for each sleep stage is listed in Table 1.

In our experiments, the 75 subjects were randomly partitioned into five groups for 5-fold cross-validation. Each group contained 30 PSG recordings from 15 subjects. This ensured that all data from the same subject did not appear in both the training and test sets. This approach helped prevent data leakage and overfitting, ensuring a fairer and more reliable evaluation of the model’s performance. Table 2 summarizes the number of 30 s epochs for each sleep stage in each cross-validation group.

### 2.2. Preprocessing

**Figure 2:**
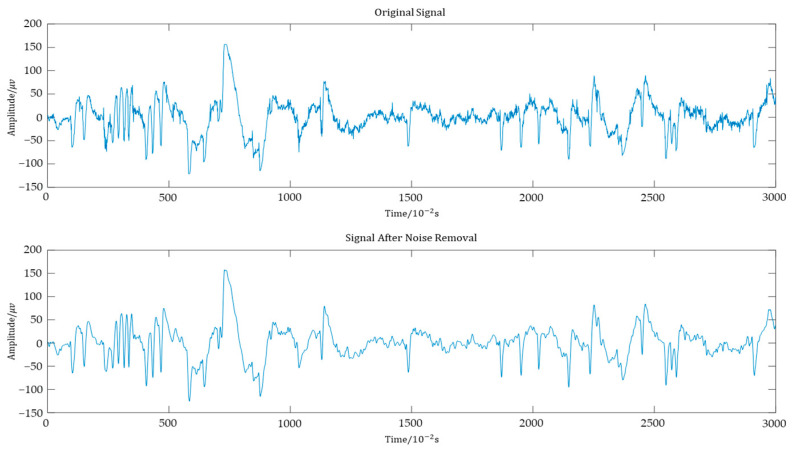
The effect of wavelet denoising on an EEG sample.

The raw data were preprocessed through wavelet thresholding denoising before feature extraction. Wavelet thresholding denoising is a widely used signal processing method for removing noise components from signals, characterized by low entropy and multi-resolution properties. This method effectively separates useful information from noise in the signal. It adapts well to the complex frequency structure of signals, preserving features across different frequency bands. We employed the Db4 wavelet basis function, commonly used for EEG signals processing, and applied an effective soft thresholding method for denoising. The Db4 wavelet provides good localization in both time and frequency domains, helping to capture instantaneous changes in EEG signals while maintaining low computational complexity. To maximize the preservation of useful signal information during denoising and accommodate the complex frequency structure of EEG signals, we chose a 7-level wavelet decomposition, followed by signal reconstruction. This multiple-level decomposition allowed us to extract features from different scales of the signal. Finally, we applied the inverse wavelet transform to reconstruct the denoised signal from the processed coefficients. An example of the wavelet denoising result is shown in Figure 2.

**Figure 3:**
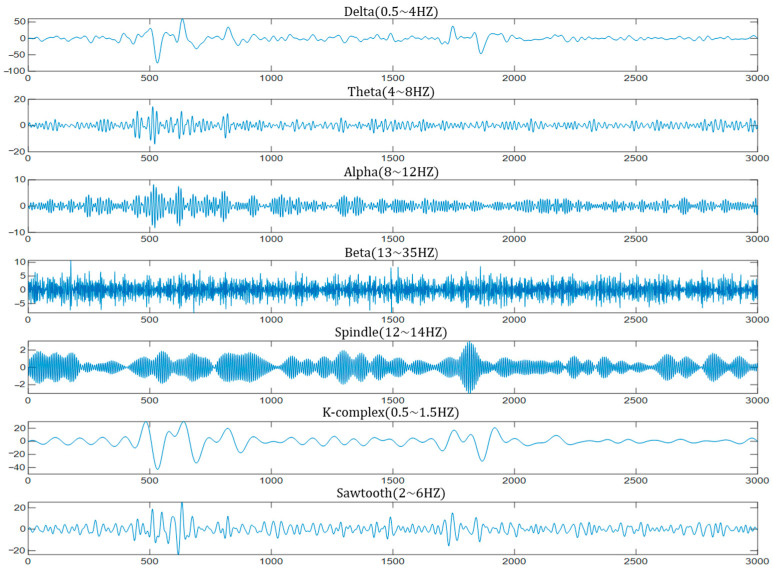
Example of filtered EEG waveforms in different frequency bands.

After denoising, an FIR digital filter with a Kaiser window was used to extract the rhythmic EEG waves. The filter separated the input signal into different frequency bands, including Delta (0.5~4 Hz), Theta (4~8 Hz), Alpha (8~12 Hz), Beta (13~35 Hz), Spindle (12~14 Hz), K-complex (0.5~1.5 Hz), and Sawtooth (2~6 Hz). We chose an FIR filter with a Kaiser window because it provides flexible frequency response characteristics, allowing for effective control over filter bandwidth and sidelobe attenuation. This design ensures that the filter adapts well to the frequency characteristics of EEG signals while minimizing signal distortion. Examples of waveforms for each frequency band obtained using FIR band-pass filters are shown in Figure 3.

### 2.3. Feature Extraction

#### 2.3.1. Time Domain Features

Seven commonly used time-domain features were extracted from the band-pass filtered EEG signals and the EOG signal, including the Range, Mean, Variance, Standard Deviation, Peak Count, Zero-crossing Count, and Difference Variance [12]. Additionally, two novel time-domain EOG features were created to improve N1 stage classification: Large Eye Movement Count [13] and Difference Variance Excluding Large Eye Movement [14].

**Figure 4:**
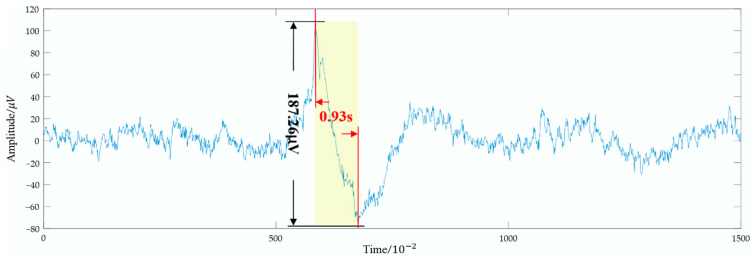
Example of a large eye movement event.

Large Eye Movement Count identifies and counts large eye movement events by analyzing the temporal characteristics of EOG signals. Based on the study by Collewijn et al. [15], time and amplitude thresholds are used to detect these events. Specifically, the amplitude threshold is set at 120 μV, and the time threshold is set at 1.5 s. When the time interval between adjacent maxima and minima is within 1.5 s and the peak-to-peak amplitude exceeds 120 μV, it is recognized as a large eye movement event, as shown in Figure 4.

**Figure 5:**
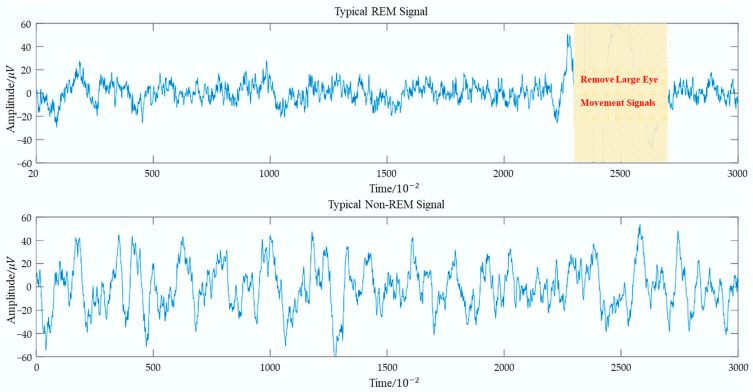
Comparison between typical REM and non-REM signals.

Difference Variance Excluding Large Eye Movement is an innovative feature that measures the smoothness of an EOG signal. As shown in Figure 5, the smoothness of EOG signals the during REM stage increases after removing large eye movement activities. To compute this feature, the detected Large Eye Movement Events and data segments that are 0.5 s before and after those events were first removed from the original EOG signal. Then, the signals went through a first-order differential filter, which calculated the difference between two adjacent data points ($D\left[n\right]=X\left[n\right]-X[n-1]$), resulting in a differential sequence. Based on this sequence, the Difference Variance ($F_{sdv}$) was computed, which is mathematically defined as follows:

(1) $$F_{sdv}=\frac{1}{N}\sum_{n=1}^{N}{\left(D\left[N\right]-\bar{D}\right)}^{2},$$

where $D\left[n\right]=X\left[n\right]-X[n-1]$ represents the first-order difference of the signal, $\bar{D}$ is the mean of the difference sequence, and N is the signal length [16].

Difference Variance reflects the degree of signal fluctuation, with smaller values indicating a smoother signal. Through extensive comparisons across numerous samples, it was observed that after removing large eye movements, the EOG signal during the REM phase became smoother than in the non-REM phase (as shown in Figure 5). This finding suggests that the fluctuations in the REM-phase signal are smaller and more stable after the removal of large eye movement events. Therefore, Difference Variance effectively captures this characteristic and serves as an indicator of signal smoothness.

#### 2.3.2. Power Spectrum Density Features

Power Spectral Density (PSD) measures how the power of the EEG signal is distributed across different frequency components. In this study, the Yule–Walker method was chosen to estimate the PSD [17] of the EEG signals, from which the Absolute Band Power Ratio and the Relative Band Power Ratio features were extracted. The Absolute Band Power Ratio is defined as the power of a frequency band divided by the total power of the EEG signal. It is computed for Delta, Theta, Alpha, Beta, K-complex, Spindle, and Sawtooth frequency bands, respectively [18].

The Relative Band Power Ratio is defined as the power ratio between two frequency bands. Specifically, the following four Relative Band Power Ratios were computed for the EEG signals.

(2) $$F_{\delta /\theta }=\frac{P([0.5,4])}{P([4,8])},$$

(3) $$F_{\theta /\alpha }=\frac{P([4,8])}{P([8,12])},$$

(4) $$F_{\alpha /\beta }=\frac{P([8,12])}{P([12,30])},$$

(5) $$F_{\left(\theta +\delta )/(\alpha +\beta \right)}=\frac{P([0.5,8])}{P([8,30])},$$

Additionally, two power ratio features, the Slow Eye Movement Ratio and the Rapid Eye Movement Ratio, were extracted from the EOG signal to distinguish the REM and W states, which are defined as follows:

(6) $$F_{slow-eye}=\frac{P([0.5,2])}{P([0.5,30])},$$

(7) $$F_{rapid-eye}=\frac{P([2,5])}{P([0.5,30])},$$

#### 2.3.3. Multiscale Entropy

Multiscale Entropy (MSE) is a method used to analyze the complexity and irregularity of time series. It calculates the sample entropy of signals at different time scales to reveal the dynamic characteristics of the signals across these scales [19]. The computation of MSE requires three parameters: the window length m, the distance threshold r, and the scale factor. To ensure that the dynamic reconstruction of joint probability contains more information, *m* was set to 2 in this study. Based on the theoretical analysis by Pincus et al. [20], when *r* is set to 0.1 SD~0.25 SD, sample entropy is more effective. Regarding the scale factor, according to Liang et al. [21], the MSE values with a scale factor between 9 and 13 are the best choices for accurately distinguishing between different sleep stages. Based on these results, *r* was set to 0.2 SD, and the scale factor was set to 12 when computing the MSE.

Table 3 summarizes all of the features extracted from the EEG and EOG signals. For each 30 s PSG epoch, the EEG signals were band-pass filtered into rhythmic waves within seven frequency bands, and each band-pass filtered EEG signal contained 7 time-domain features, 11 PSD features, and 5 MSE features. In addition, the EOG signal contained 9 time-domain features and 2 PSD features. These together resulted in a comprehensive multi-domain, multi-modal set of 76 features.

### 2.4. Feature Selection

To reduce the computational cost during the classification stage and identify the most discriminating information for identifying different sleep stages, we propose a two-step feature selection strategy that employs the Fisher Score for pre-filtering, followed by XGBoost feature importance ranking.

Fisher Score is an effective filter-based approach for feature importance analysis. The main idea is that features with strong discriminative performance have a small within-class distance and a large between-class distance [22]. *x*(*k*) denotes the value of sample *x* on the *k*-th feature, $m_{i}^{\left(k\right)}$ represents the mean value of the *k*-th feature for the samples in the *i*-th class, and *m*^(*k*)^ represents the mean value of the *k*-th feature for all classes. The between-class variance of the *k*-th feature in the dataset is defined as $S_{B}^{\left(K\right)}$, given by the following equation:

(8) $$S_{B}^{\left(K\right)}=\sum_{i=1}^{C}\frac{n_{i}}{n}{\left(m_{i}^{\left(k\right)}-m^{\left(k\right)}\right)}^{2},$$

The within-class variance of the *k*-th feature in the dataset is defined as $S_{w}^{\left(K\right)}$, given by the following equation:

(9) $$S_{W}^{\left(K\right)}=\frac{1}{n}\sum_{i=1}^{C}\sum_{x\in \omega_{i}}{\left(x^{\left(k\right)}-m_{i}^{\left(k\right)}\right)}^{2},$$

Finally, the Fisher Score of the k-th feature in the dataset, denoted as $J_{fisher}^{\left(k\right)}$, is defined as follows:

(10) $$J_{fisher}^{\left(k\right)}=\frac{S_{B}^{\left(K\right)}}{S_{w}^{\left(K\right)}},$$

**Figure 6:**
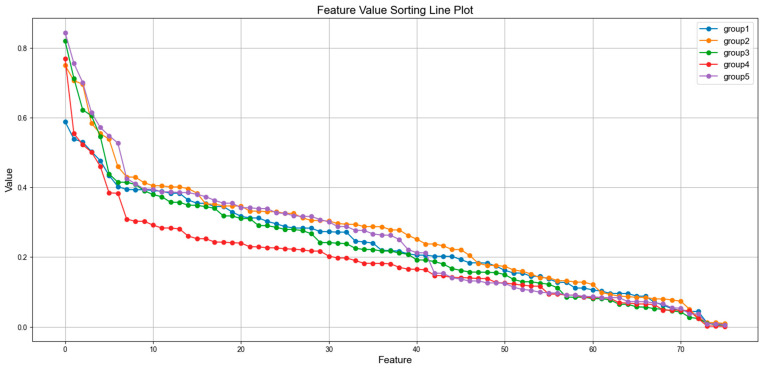
Fisher Score of all features in each cross-validation fold.

In Figure 6, we show the Fisher Score of all features for each cross-validation fold in descending order. As a univariate method, Fisher Score quickly identifies features with low discriminating power. In our experiments, features with a Fisher Score below 0.1 were discarded, while the remaining features with a Fisher Score higher than 0.1 were kept for next step feature ranking. This two-step strategy helped us focus on the most informative features for further analysis, ensuring that only the most relevant features proceed to the next stage of feature selection.

The Fisher Score can effectively and quickly filter out features with low discriminating power. However, as a univariate approach, it does not measure the combined effect of different features. Therefore, in the second step, the F-score filtered feature subset was fed into an XGBoost model for feature importance analysis. XGBoost is an efficient gradient boosting decision tree (GBDT) method that is widely used in multi-classification tasks [23]. It improves prediction accuracy by integrating multiple weak classifiers, usually decision trees. In the XGBoost model, feature importance is calculated for a single decision tree by the amount that each attribute split point improves the performance measure, weighted by the number of observations the node is responsible for. The feature importance scores are then averaged across all of the decision trees within the model to obtain the final feature importance score.

**Figure 7:**
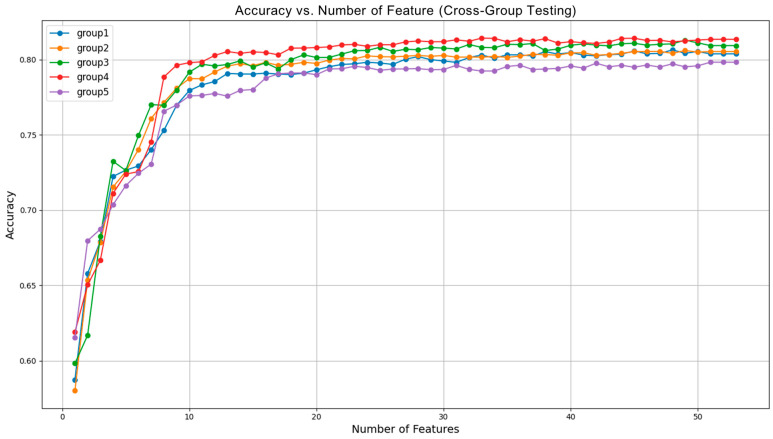
Accuracy vs. number of features (cross-group testing).

In Figure 7, we show the classification accuracy versus the different number of features in each cross-validation fold. From the figure, we can see that the classification performance became stabilized when the feature number reached 25. This suggests that selecting 25 features strikes a good balance between maintaining high classification accuracy and reducing the number of features used. This further supports the feasibility of our second-step feature selection method, which helps optimize performance while minimizing complexity.

### 2.5. Classification Model

After feature selection, an XGBoost classifier was trained based on the selected features for sleep stage classification. XGBoost has several advantages for multi-classification tasks: (1) it can handle nonlinear data and high-dimensional features; (2) it employs regularization techniques to help prevent overfitting; (3) it supports missing value handling and the automatic encoding of categorical features; and (4) its high computational efficiency allows it to handle large-scale datasets [24].

In this study, hyperparameter tuning for XGBoost was performed using a grid search. Within each fold of training data, 80 PSGs were used as the learning set for parameter tuning and 40 PSGs were used for validation. The optimal parameter combination was as follows: colsample_bytree = 0.8, learning_rate = 0.2, max_depth = 7, n_estimators = 300, reg_alpha = 0.1, reg_lambda = 1, and subsample = 0.8. Subsequently, these 120 PSGs were combined to train the XGBoost model with optimal parameters for sleep stage classification on the test data.

## 3. Experiments and Results

### 3.1. Evaluation Methods

In the experiments, independent training and testing sets were used for model evaluation, i.e., all data from the same subjects were either in the training set or the test set. This ensured an accurate evaluation of the generalization ability of the proposed methods. For each cross-validation fold, the training set, which consisted of 60 subjects, was further divided into a learning set of 40 subjects and a validation set of 20 subjects for XGBoost parameter tuning and feature selection. With the optimized parameters and feature set, an XGBoost model was trained on the entire training set to classify the testing set.

Several widely used metrics are used to assess classification performance, including Accuracy, F1-score the Kappa coefficient (κ), Precision (PR), and Recall (RE) [25,26]. The definitions of these metrics are as follows:

(11) $$Accuracy=\frac{TP+TN}{TP+FP+TN+FN},$$

(12) $$Precision=\frac{TP}{TP+FP},$$

(13) $$Recall=\frac{TP}{TP+FN},$$

(14) $$F1=2*\frac{Precision*Recall}{Precision+Recall},$$

(15) $$\kappa =\frac{p_{o}-p_{e}}{1-p_{e}},$$

where TP (True Positive) refers to the correctly predicted positive cases, TN (True Negative) refers to the correctly predicted negative cases, FP (False Positive) refers to the incorrectly predicted positive cases, and FN (False Negative) refers to the incorrectly predicted negative cases. $p_{o}$ represents Accuracy, and $p_{e}$ is the sum of the product of actual quantities and predicted quantities divided by the square of the total sample size.

### 3.2. Classification Results

Table 4 shows the overall confusion matrix obtained from the 5-fold cross-validation using the top 25 features. Each row and column represents the number of epochs classified by the sleep experts and our model, respectively. The numbers in bold indicate the number of epochs that were correctly classified. We can see that the Wake stage is most distinguishable from other stages, while the N1 stage, with an F1 score of less than 50, shows the poorest performance. A significant number of N1 (light sleep) epochs were misclassified into the N2 (intermediate sleep) stage, while the misclassification between the REM and Wake stages also occurred. These findings are consistent with existing sleep staging works, including state-of-the-art deep learning methods.

In Table 5, we present the classification results using different feature sets to show the effect of feature engineering and feature selection on sleep staging performance. The overall classification accuracy was 84.4% when only EEG features were used, while the result improved to 87.5% after adding EOG features. In particular, the performance of the N1 (F1 from 44.3 to 53.3) and REM (F1 from 77.5 to 84.1) stages improved significantly with the help of the EOG features. This result indicates that eye movement activity patterns play a critical role in distinguishing different sleep stages.

We next compared the classification results using a different number of features. As expected, the best classification performance was achieved when all 76 features were included, which indicates the expressiveness of our comprehensive feature set. A very close performance was achieved using the 25 top features after feature selection. However, when further reducing the feature number to 10, the classification performance dropped significantly, especially in the hard-to-distinguish N1 and REM stages. These results together demonstrate that our feature selection algorithm can effectively select a subset of discriminating features and greatly reduce the computational complexity in the prediction phase.

### 3.3. Feature Analysis

**Figure 8:**
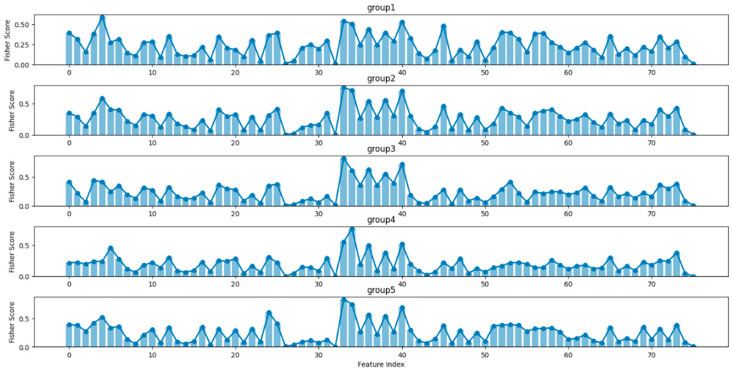
Fisher Score of all features in each fold.

**Figure 9:**
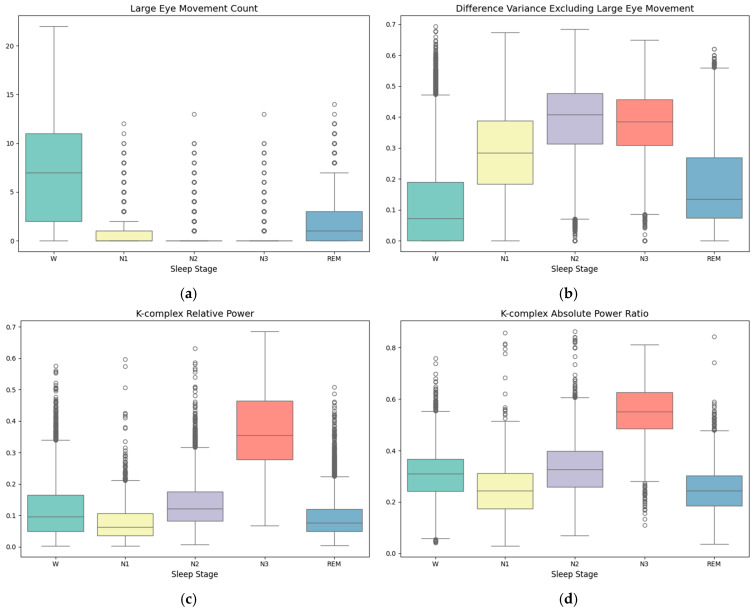

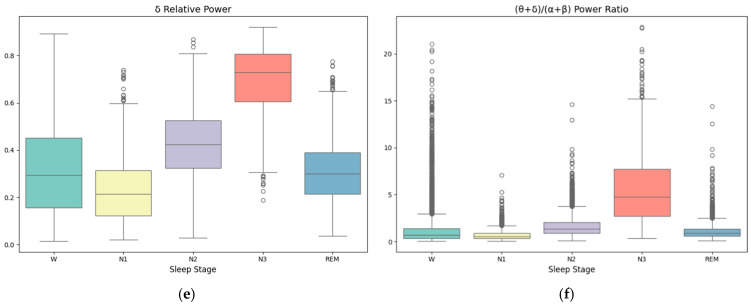
Boxplot of top features. (**a**) Large Eye Movement Count, (**b**) Difference Variance Excluding Large Eye Movement, (**c**) K-complex Relative Power, (**d**) the K-complex Absolute Power Ratio, (**e**) the α/β Power Ratio, and (**f**) the (θ + δ)/(α + β) Power Ratio.

In Figure 8, we show the F-score of all 76 features within each cross-validation fold and analyze those with the highest scores to learn the discriminative information between different sleep stages, including Large Eye Movement Count (33), Difference Variance Excluding Large Eye Movement (34), K-complex Relative Power (38), K-complex Absolute Power Ratio (40), α/β Power Ratio (3) and (θ + δ)/(α + β) Power Ratio (4). Note that these features were consistently ranked with high feature importance by XGBoost in each cross-validation fold. The boxplots of these top features are shown in Figure 9. It is important to note that the dots in Figure 9 represent outliers, which are caused by the characteristics of individual samples or noise factors. These outliers are not discussed here, as we focus on the values of the majority of the samples.

As shown in (Figure 9a), the Large Eye Movement Count feature exhibits significantly higher values during the Wake and REM stages, followed by N1 (light sleep), while the values are lowest during N2 (intermediate sleep) and N3 (deep sleep). As shown in (Figure 9b), the Difference Variance Excluding Large Eye Movement feature has higher smoothness and higher values in N2 and N3, with slightly lower values in N1. This is consistent with the characteristics of eye movement activity across different sleep stages, aligning with the regular patterns of eye movement in each stage and helping to distinguish N1 from other stages.

As shown in Figure 9c,d, both the K-complex Relative Power feature and the K-complex Absolute Power Ratio feature reach their highest values during N3, followed by N2, while the values are lower during the Wake and REM stages. This aligns with the physiological characteristic of K-complexes predominantly occurring in N2, making these features essential for identifying the N2 stage.

As shown in Figure 9e,f, the δ Relative Power feature and the (θ + δ)/(α + β) Power Ratio feature are lower during N1 and N2 but significantly increase during N3. This is consistent with the dominance of δ waves during N3, aligning with the EEG spectral distribution characteristics of different sleep stages and highlighting the significance of these features in identifying the N3 stage.

## 4. Discussions

To further demonstrate the effectiveness of the proposed method, we performed a comprehensive comparison analysis with state-of-the-art sleep staging methods that use the same Sleep-EDF dataset, as shown in Table 6. Multiple dimensions were analyzed, including signal channel selection, the number of signal frames used for testing, the number of features, overall classification performance, and the performance for each stage. This multi-dimensional comparison allowed for a more thorough evaluation of the performance of different methods under varying conditions.

In related works, the classification methods can be divided into two categories: non-independent training and testing set methods, and independent training and testing set methods. Non-independent methods are characterized by their inclusion of part of the test data during the training process, which may lead to data leakage during testing, thus affecting the accuracy of the evaluation. In contrast, independent training and testing set methods ensure complete independence between the training and testing sets, with no overlap of test data during training, providing a more accurate reflection of the model’s generalization ability. In practice, evaluation schemes must ensure the independence of the testing set to avoid overfitting and ensure the reliability of the evaluation results. Therefore, this study emphasizes the importance of using independent training and testing sets, as it helps to improve the model’s performance and stability in real-world applications and provides a more stringent evaluation standard for future research.

The proposed sleep staging method achieved a competitive overall classification accuracy of 87%, with an F1 score of 86.6 and a κ value of 0.81. In particular, our method achieved the best performance in the hardest N1 stage compared to previous methods that use independent training and test sets, including state-of-the-art deep learning methods.

In feature selection, a two-step feature selection algorithm was employed that significantly decreased the computational complexity of the model. Through preliminary feature selection with the Fisher Score and secondary screening using XGBoost, the dimensionality of the features was significantly reduced while maintaining satisfactory classification performance. This process effectively reduced the risk of overfitting and enhanced the model’s robustness.

Additionally, this study extracted two novel EOG features, “large eye movement detection” and “variance difference after removing large eye movements”, which significantly improved classification accuracy. These features demonstrated a strong discriminatory ability to improve the classification accuracy for the N1 stage. Features from different frequency bands, including low and high frequencies, also showed varying effects, further proving the effectiveness of multi-frequency features in sleep staging. Although the classification accuracy for the N1 stage improved, it still faced challenges compared to other sleep stages.

Overall, the classical machine learning pipeline typically consists of the following steps: data collection, data preprocessing, feature extraction, feature selection, model training and validation, and model evaluation. Compared to the traditional machine learning pipeline, our approach integrates features from multiple domains in the feature extraction step, while also demonstrating higher efficiency and accuracy in feature selection and classification performance. This is particularly evident when handling multi-domain features and complex signals.

## 5. Conclusions

In this paper, we proposed an effective and interpretable sleep stage classification scheme that follows the classical machine learning pipeline. Multi-domain features were extracted from preprocessed frontal EEG and EOG signals, including novel eye movement features that significantly improved N1 stage classification accuracy. A two-step feature selection strategy combining F-score prefiltering and XGBoost feature importance ranking was designed to select the most discriminating feature subset, which significantly reduced the feature dimensionality for prediction while maintaining high discriminating power. This also laid the foundation for further experimentation with more new features in the future. Through a rigorous double-cross-validation procedure, our approach achieved competitive classification performance using XGBoost on the public Sleep-EDF dataset compared to state-of-the-art deep learning methods. In addition, feature importance analysis provided knowledge about the characteristics of different sleep stages. These promising results demonstrate the effectiveness of the proposed sleep staging model and show its potential in practical applications due to its low complexity, interpretability, and transparency. Looking ahead, future research will focus on the following directions:

1. Developing more effective feature sets, particularly for the N1 stage, to further improve classification accuracy.
2. Exploring more advanced feature selection algorithms to enhance the accuracy and adaptability of feature selection.
3. Designing low-complexity, high-accuracy, and interpretable classification models to optimize N1 stage classification.
4. Testing the models on more diverse datasets to validate their stability and adaptability across different populations and environments.

## Figures and Tables

**Table 1:** Number of 30 s epochs for each sleep stage.

Label	W	N1	N2	N3	REM
Number of Frames	52,492	15,064	60,473	8203	23,364

**Table 2:** Data statistics for 5-fold cross-validation.

Label	W	N1	N2	N3	REM	Total
Part 1	9752	2808	12,430	1837	4648	31,475
Part 2	10,900	3712	11,390	2128	4640	32,770
Part 3	9652	3192	11,483	1488	4582	30,397
Part 4	12,447	2561	12,525	1376	4699	33,608
Part 5	9741	2791	12,645	1374	4795	31,346
Total	52,492	15,064	60,473	8203	23,364	159,596

**Table 3:** List of all features.

Function	EEG	#Features	EOG	#Features
**Time-domain**				
Range, Mean, Variance, Standard Deviation, Peak Count, Zero-crossing Count, Difference Variance	√	49	√	7
Large Eye Movement Detection Difference Variance Excluding Large Eye Movement	-	-	√	2
**Power Spectrum Density**				
Absolute power ratios of different frequency bands (Delta, Theta, Alpha, Beta, K-complex, Spindle and Sawtooth)	√	7	-	-
Spectral Power Ratio: $F_{\delta /\theta },F_{\theta /\alpha },F_{\alpha /\beta },F_{\left(\theta +\delta )/(\alpha +\beta \right)}$	√	4	-	-
Eye movement power ratio: $F_{slow-eye},F_{rapid-eye}$	-	-	√	2
**Multiscale Entropy**				
Sample Entropy	√	5	-	-

**Table 4:** Confusion matrix and per-class performance metrics using top 25 features.

	Predicted					Per-Class Metrics		
	W	N1	N2	N3	REM	PR	RE	F1
W	49,878	1371	629	12	781	92.68	94.63	93.64
N1	1865	6178	4688	14	1575	57.30	42.37	48.54
N2	884	1998	56,742	750	1768	86.62	91.28	88.85
N3	120	3	1182	6803	6	88.88	83.33	85.98
REM	1070	1198	2223	17	17,840	81.22	79.87	80.54

**Table 5:** Performance comparison with different feature sets.

Method	Per-Class F1-Score					Overall Metrics		
	W	N1	N2	N3	REM	Accuracy	MFI	κ
67 features (EEG only)	92.0	43.3	88.2	85.9	77.5	84.4	83.8	0.78
76 features (EEG + EOG)	94.8	53.3	89.9	87.4	84.1	87.5	87.1	0.82
25 features (EEG + EOG)	93.6	48.5	88.9	86.0	80.5	87.0	86.6	0.81
10 features (EEG + EOG)	90.4	36.1	86.1	80.3	69.3	83.0	82.3	0.76

**Table 6:** Comparison of classification methods based on multiple criteria.

Methods	EEG Channel	Test Epochs	Feature Count	Overall Metrics			Per-Class F1-Score(F1)				
				ACC	MFI	κ	W	N1	N2	N3	REM
Non-independent Training and Test Sets											
Ref. [27]	Fpz-Cz	960	-	90.3	76.5	-	77.3	46.5	94.9	72.2	91.8
Ref. [28]	Pz-Oz	15,136	50	91.3	77	0.86	97.8	30.4	89	85.5	82.5
Ref. [29]	Pz-Oz	7596	-	90.8	80	0.85	96.9	49.1	89	84.2	81.2
Independent Training and Test Sets											
This paper	Fpz-Cz EOG	159,596	25	87.0	86.6	0.81	93.6	48.5	88.9	86.0	80.5
Ref. [30]	Fpz-Cz	37,022	35	78.9	73.7	-	71.6	47.0	84.6	84.0	81.4
Ref. [31]	Fpz-Cz	37,022	35	74.8	69.8	-	65.4	43.7	80.6	84.9	74.5
Ref. [32]	F3-M2 F4-M1	-	62	77.0	-	-	84.6	31.1	77.8	85.3	75.4
Ref. [7]	Fpz-Cz	32,485	-	84.2	75.3	0.78	86.7	33.2	87.1	87.1	82.1
Ref. [6]	Fpz-Cz C4-A1	41,950	-	82.0	76.9	0.76	84.7	46.6	85.9	84.8	82.4
Ref. [6]	Pz-Oz	41,950	-	79.8	73.1	0.72	88.1	37	82.7	77.3	80.3

## Data Availability

The original data presented in the study are openly available from PhysioBank at https://physionet.org/content/sleep-edfx/1.0.0/ (accessed on 11 March 2025).
